# Sleep health challenges among women: insomnia across the lifespan

**DOI:** 10.3389/frsle.2024.1322761

**Published:** 2024-02-12

**Authors:** Elizabeth Benge, Milena Pavlova, Sogol Javaheri

**Affiliations:** Division of Sleep and Circadian Disorders, Brigham and Women's Hospital and Harvard Medical School, Boston, MA, United States

**Keywords:** insomnia, menopause, women, pregnancy, gender discrimination

## Abstract

The presentation of sleep disorders varies widely among women and men, and sleep disorders among women are frequently subject to under- and delayed diagnosis. Insomnia is a complex sleep disorder with a multifactorial etiology, and women face many sex-specific sleep health challenges that may contribute to and influence the presence of insomnia symptoms across their lifespan. These include sex differences in neurobiology, hormonal variation during menstruation, pregnancy and menopause, increased prevalence of mood disorders, increased vulnerability to adverse socioeconomic factors, and gender discrimination, among other psychosocial stressors, particularly among women of racial-ethnic minority. As the medical community continues to recognize the significance of sleep as a vital pillar of overall wellbeing, the integration of sex-specific considerations in research, diagnosis, and treatment strategies is essential to optimizing sleep health for women.

## Introduction

Sleep is essential for our physical and mental health. It has a profound influence on various aspects of our lives, affecting cognitive function, emotional balance, and overall wellbeing. While the significance of sleep health holds true for all individuals, emerging evidence underscores its specific importance for women. Inadequate sleep has been correlated with negative health consequences in adults, particularly among women (Vézina-Im et al., 2017). Compounding the problem, sleep disorders in women are frequently subject to delay and underdiagnosis (Marsella and Sharkey, 2020). Additionally, the presentation of sleep disorders in women can differ compared to men and sleep changes throughout the lifespan of an adult woman (Soares and Murray, 2006). This review explores some of the sleep health challenge in women across the lifespan with a special focus on insomnia. The purpose of this review is to raise awareness about some of the unique challenges women face in the realm of sleep health and to provide a general framework for understanding the prevalence and social dynamics of sleep disorders, particularly insomnia, among women.

## Insomnia

Insomnia, broadly characterized as a difficulty in initiating or maintaining sleep coupled with a daytime impairment, is a disease process with a notably higher prevalence in older adults and women (Roth, 2007; Low et al., 2020). Correspondingly, women are more likely to acknowledge and report insomnia-related symptoms to their healthcare providers as compared to men (Hale et al., 2009). A recent meta-analysis assessing insomnia prevalence from the general population pooled data from 13 studies and found that women had a significantly higher prevalence of insomnia than men (OR = 1.58, 95% CI: 1.35, 1.85, *p* < 0.0001) (Zeng et al., 2020).

The potential mechanisms underlying the increased risk of insomnia in women are manyfold including sex differences in neurobiology, sex steroids including hormonal variation during menstruation, pregnancy and menopause, increased prevalence of mood disorders among women, environmental factors, increased vulnerability to adverse socioeconomic factors, and other psychosocial stressors.

Women may experience greater levels of pre-sleep arousal, with resultant difficulties in falling asleep. This may be attributed in part to higher reports of worrying around bedtime in women as compared to men (El Rafihi-Ferreira et al., 2022). Working middle-aged women report ruminating and worrying about daily tasks at night, and may be under increased stress induced by the competing demands of paid work and unpaid domestic labor which may also restrict time that can be allocated toward adequate sleep (Sidani et al., 2019). The higher prevalence of affective disorders among women also likely contributes to these gender disparities in insomnia (Hale et al., 2009). While insomnia and mood disorders are distinct but overlapping entities, patients with anxiety and depression are more prone to developing insomnia (Grewal and Doghramji, 2017). Women are two to three times as likely to be diagnosed with depression and/or anxiety over the course of their lifetime when compared to men (Pigott, 1999; Wang et al., 2021). Women are also at risk of more specific types of depression-related illnesses such as premenstrual dysphoric disorder, postpartum depression, and postmenopausal depression and anxiety (Albert, 2015; Wang et al., 2021). Biological factors, such as polymorphisms in the serotonin-linked polymorphic region (5HTTLPR) and an inherited increased susceptibility to hormonal changes may also play a role in the depression and anxiety gender gap (Vigod and Stewart, 2009). Other hormonal differences, such as the variation in ovarian hormone levels and, particularly, decreases in estrogen, may contribute to the increased prevalence of depression and anxiety in women (Vigod and Stewart, 2009).

In addition to contributing to insomnia directly, other factors such as gender-specific cultural and social roles as well as gender discrimination may also contribute to the female preponderance of depression and anxiety. A survey by the World Health Organization (WHO) found that more than one in ten women endorse experiencing perceived gender discrimination (Takács et al., 2016). Experiencing perceived gender discrimination was significantly associated with higher depression scores even after adjusting for poverty, social support, and gender inequality (Takács et al., 2016). Worldwide, research has also shown that societal disparities, such as economic reliance, limited decision-making authority, contradictory gender expectations, unequal distribution of domestic duties, and instances of violence that women bear disproportionately to men are a key driver behind the psychological stress that precipitates the development of mental health disorders (Vigod and Stewart, 2009, Chiu et al., 2021; Wang et al., 2021). Given the bidirectional relationship between insomnia and mental health disorders, the unequal burden of depression and anxiety in women likely accounts for some of the gender-based variations in insomnia prevalence (Netzer et al., 2003).

### Pregnancy

Throughout pregnancy, sleep trends progress from hypersomnolence in the early trimesters to insomnia in the third trimester and postpartum (Lee, 1998; Meers and Nowakowski, 2022). The first trimester is characterized by poor sleep quality and heightened sleepiness compared to non-pregnant individuals (Okun et al., 2015). In the third trimester, having <6 h of objective nighttime sleep is linked to a notable risk of clinical depression in healthy expectant mothers (Tsai et al., 2016). These findings underscore the significant interplay between sleep patterns, pregnancy, and mental wellbeing, highlighting the importance of comprehensive care strategies for sleep health during pregnancy.

Pregnant women may encounter a range of sleep disorders throughout pregnancy that can also contribute to insomnia symptoms including obstructive sleep apnea (OSA) and restless leg syndrome (RLS) (Meers and Nowakowski, 2022). The prevalence of OSA among pregnant women is 15% and presence of OSA, in addition to contributing to insomnia symptoms, is also associated with maternal hypertension, gestational diabetes, and preterm birth (Meers and Nowakowski, 2022). Subjectively, pregnant women report experiencing significant sleep disruption, inadequate sleep, and high rates of sleep disorder symptoms throughout their pregnancies (Mindell et al., 2015). The high prevalence of pregnancy-related sleep dysfunction, and its correlation to poor health outcomes, emphasizes the need for targeted interventions and support for sleep disorders during pregnancy.

RLS is a sleep disorder commonly diagnosed in pregnant women; up to 20% of women may experience RLS during pregnancy and its presence has been associated with insomnia and depression among other adverse outcomes (Jahani Kondori et al., 2020, Meers and Nowakowski, 2022). Symptoms typically present in the second or third trimester and it is typically a transient condition with the majority of cases resolving after delivery or by ~1 month postpartum (Meharaban et al., 2015; Prosperetti and Manconi, 2015). It is highly associated with iron deficiency anemia and, to a lesser extent, smoking (Tunç et al., 2007; Meharaban et al., 2015; Naik and Polen, 2020) though there are additional potential etiologies including genetic mechanisms, estrogen deficiency, and stretch of nerves during pregnancy (Meharaban et al., 2015). First line treatment options include gentle exercise, such as yoga (Muntean et al., 2021). When clinically appropriate, iron replacement is also an effective therapy though pregnant women may have difficulty tolerating oral iron therapy. In severe cases, additional pharmacotherapy may be considered after the first trimester on a case by case basis (Meharaban et al., 2015; Silber et al., 2021). Increased screening for and education regarding RLS during pregnancy is one potential area to target improved sleep health among women and may also help improve insomnia symptoms among pregnant women.

#### Perimenopause

Research findings highlight distinct sleep patterns across the menstrual cycle with additional variation pre- and perimenopause. A study analyzing data from a single menstrual cycle of 630 women sought to determine whether hormone levels were associated with difficulty sleeping and compared differences between perimenopausal and premenopausal women. The adjusted odds of sleep disturbance were 29% higher in perimenopausal than premenopausal women. Both groups reported highest rates of difficulty sleeping during the initial and final stages of the menstrual cycle. Additionally, mood and vasomotor symptoms were most robustly associated with difficulty sleeping. Finally, follicle-stimulating hormone levels were associated with increased difficulty sleeping in premenopausal women, while pregnanediol glucuronide (a progesterone metabolite) level was associated with increased difficulty sleeping in peri-menopausal women (Kravitz et al., 2005). This study highlights the complex relationship between hormonal changes and sleep quality throughout the menstrual cycle, and that difficulty sleeping occurred most prominently at the beginning and end of menstruation.

#### Menopause

Approximately half of all women (40–56%) report sleep difficulties peri- and post-menopause (Carmona et al., 2023). In a multiethnic cohort of pre or peri-menopausal women (*N* = 3,302), insomnia symptoms were present in 31–42% of perimenopausal women at any 1-year study interval across a span of 10 years (Ciano et al., 2017). Insomnia symptoms were more prevalent in the late stage of perimenopause than the early stage and up to 26% of women develop chronic clinical insomnia (severe sleep symptoms coupled with daytime impairment) after menopause (Kravitz et al., 2003; Baker et al., 2018). Sleep difficulties are uniquely tied to menopause and the fluctuations in follicle-stimulating hormone (FSH) and estradiol levels experienced during this time (Baker et al., 2018). Increasing FSH is associated with increased odds of sleep fragmentation while decreased estrogens have been associated with increased odds of difficulty falling and staying asleep in the SWAN study (Kravitz et al., 2003). In addition to hormonal changes during menopause, vasomotor symptoms such as hot flashes and sweating, headaches, genitourinary symptoms, headaches, and mood symptoms may also contribute to insomnia symptoms specific to menopause (Kravitz et al., 2003; Carmona et al., 2023).

Further compounding this is the multifold increase in OSA in close relation to menopause even in the absence of clinical complaints (Bonsignore et al., 2019). The risk of developing sleep apnea in women increases significantly after menopause, though the etiology of this is complex and continues to be poorly understood (Bixler et al., 2001; Bouloukaki et al., 2021). However, in women OSA may present differently than in men and frequently includes insomnia or sleep fragmentation, which can be clinically attributed to other causes, thus delaying testing and diagnosis, since half of women in this age group report insomnia (Suzuki et al., 2017).

In terms of treatment, the research regarding the impact of hormonal replacement therapy on sleep quality is mixed. No significant difference in sleep efficiency was found in estrogen replacement and placebo groups in one study (Tansupswatdikul et al., 2015). Conversely, estrogen replacement therapy was found to enhance objective sleep quality by reducing the frequency of nocturnal movement arousals in another study (Polo-Kantola et al., 1999). Data on the effect of hormone replacement therapy on sleep disordered breathing is also insufficient to yet draw any meaningful conclusions on potential effect of HRT on sleep apnea post menopause (Lindberg et al., 2020). Larger RCTs in women are needed to understand the role of HRT as a therapeutic target of sleep disorders in postmenopausal women.

On the contrary, cognitive behavioral therapy for insomnia (CBT-I) has generally proven to be efficacious insomnia treatment among peri and postmenopausal women (Carmona et al., 2023). Additionally, a variety of alternative, non-pharmacologic treatments for insomnia in adults have been explored, with varying levels of evidence supporting their effectiveness. One study examined, among other treatments, the impact of acupuncture and repetitive transcranial magnetic stimulation (rTMS), on sleep quality and insomnia severity and found some benefit (Ell et al., 2023). Yoga was found to positively influence sleep symptoms in an elderly population of insomnia patients (Gulia and Sreedharan, 2021). Ultimately more studies are needed, however. If non-pharmacological approaches prove insufficient, pharmacological options may be considered, including sedative-hypnotic medications (Sweetman et al., 2021). The choice of treatment should be guided by a thorough assessment of the patient's medical history, preferences, and the severity of insomnia. Personalized and holistic approaches that consider both behavioral and pharmacological elements are crucial for effective insomnia management. Regular monitoring and adjustments to the treatment plan may be necessary to optimize outcomes and address any potential side effects.

### Sleep disorders and gynecological conditions

#### Sleep implications of gynecological conditions

Sleep problems are common in women with conditions like endometriosis and polycystic ovary syndrome (PCOS) (Singh et al., 2014). Endometriosis is a complex and often debilitating gynecological disorder characterized by the presence of endometrial-like tissue outside the uterine cavity (Eskenazi and Warner, 1997). It is also highly associated with disordered sleep. In a survey of 275 women with endometriosis, poorer overall quality of sleep was independently associated with poorer functional quality of life (EHP-30) (*b* = −0.18, *p* = 0.0026), more depressive symptoms (PHQ-9) (*b* = −1.62, *p* < 0.001), and painful bladder syndrome (PBS) (*b* = −5.82, *p* = 0.035) (Arion et al., 2020). A cross-sectional study that included women with endometriosis used the Pittsburgh Sleep Quality Index (PSQI) to quantity the relationship between endometriosis and disordered sleep, any score over 5 on this survey is indicative of poor sleep. The mean PSQI score was 6.1 ± 3.4, and 54.1% of the endometriosis patients surveyed had a PSI score over 5, demonstrating a high prevalence of sleep dysfunction in this population (Davari-Tanha et al., 2014). Another survey aimed at elucidating the effect of sleep disturbances on quality of life in women with endometriosis found that patients with comorbid endometriosis and sleep dysfunction experienced a higher burden of dysmenorrhea, pelvic pain, and dyspareunia (Youseflu et al., 2020).

PCOS is another common endocrine disorder characterized by hormonal imbalances, metabolic disturbances, and reproductive dysfunction (Carmina and Lobo, 1999). It is also associated with increased sleep disturbances, thought to be caused by the elevated levels of nighttime melatonin and 8-hydroxy-2-deoxyguanosine associated with the disease (Shreeve et al., 2013; Sam and Ehrmann, 2019). A cohort study examining the relationship between risk and severity of obstructive sleep apnea (OSA) and glucose metabolism in PCOS found that 62.5% of the women surveyed had poor sleep quality by Pittsburgh Sleep Quality Index, and 18 (45%) had chronic daytime sleepiness by Epworth Sleepiness Scale (Tasali et al., 2006). Another cross-sectional study found that, even when controlling for weight and depressive symptoms, sleep dysfunction is almost twice as common in women with PCOS compared to women of similar age without PCOS (Moran et al., 2015). These findings were replicated in a longitudinal study with 6,578 participants, which found that women with PCOS had similar sleep duration but were more likely to have trouble sleeping often compared to women without PCOS (RRR 1.67, 1.20–2.33, *P* = 0.003). This study also controlled for BMI, depressive symptoms, demographic and comorbid factors (Mo et al., 2019).

### Sleep health disparities among racial-ethnic minority women

Racial/ethnic minority women are disproportionately affected by poor sleep health compared to white women and their male counterparts. Black and Hispanic/Latina women exhibit a higher prevalence of insomnia, insufficient sleep with insomnia symptoms, inconsistent sleep schedules, increased sleep debt, and more frequent napping compared to white women (Jackson et al., 2020a,b).

In addition to increased insomnia, African-Americans are also more likely to experience sleep-disordered breathing/obstructive sleep apnea, reduced slow-wave sleep (a restorative stage of sleep associated with reduced blood pressure) (Javaheri et al., 2018), and a greater burden of physical fatigue compared to white women (Thomas et al., 2006; Ruiter et al., 2010; Grandner et al., 2013). A multitude of factors may contribute to these disparities, including but not limited to ethnic discrimination, housing type (Johnson et al., 2019; Jackson et al., 2020a,b), and traumatic childhood experiences (McWhorter et al., 2019; Jackson et al., 2020a,b). Adverse early-life exposures such as sexual, physical, and emotional traumas, natural disasters, household dysfunction, and major accidents may all fall under the category of traumatic childhood experiences and are associated with sleep disturbances in adulthood (McWhorter et al., 2019). Given mounting evidence of sleep health disparities that may be associated with wide-ranging adverse health outcomes among African-American and Hispanic/Latina women, there is a critical need for tailored therapy to higher risk groups in sleep medicine (Jackson et al., 2020a,b).

In addition to the needs for additional research to address the complicated public health challenges faced by ethnic/racial minority women, clinical providers can provide culturally competent medical care by developing an understanding of cultural norms and practices that might impact the healthcare decisions of their patients and screening for social/environmental factors that may adversely affect sleep health (Teal and Street, 2009). Incorporating cultural humility and empathy helps build trust and rapport in traditionally under-served populations (Cobb, 2010). Tailoring treatment plans to accommodate cultural preferences and beliefs, while ensuring evidence-based care, enhances patient engagement and improves health outcomes. Collaborating with interpreters or cultural liaisons when necessary and staying informed about cultural competence best practices contributes to providing effective and respectful medical care for patients from diverse backgrounds.

### Addressing sleep disparities in lesbian, gay, bisexual, transgenter, queer+ (LGBTQ+) populations

LGBTQ+ adolescents are more likely to report short sleep duration in comparison to their heterosexual peers (Dai et al., 2019). This discrepancy is influenced by various factors including demographics, substance use, and experiences of victimization or mental health challenges (Dai et al., 2019). LGBTQ+ adults also have a higher likelihood of experiencing disordered sleep (Patterson and Potter, 2020). Bisexual adults have a 1.4 times greater relative risk of developing sleep difficulties compared to heterosexual adults (Duncan et al., 2018).

Experiencing daily discrimination is linked to poorer sleep outcomes, including shorter sleep onset latency, increased sleep disturbance, heightened daytime dysfunction, and greater daytime sleepiness (Yip et al., 2020). Moreover, queer, questioning, and transgender individuals might encounter more significant healthcare obstacles compared to their lesbian, gay, and cisgender counterparts (Macapagal et al., 2016). Notably, transgender and gender-diverse participants face higher levels of discriminatory experiences and increased barriers to accessing care, indicating the importance of targeted interventions to improve their healthcare experiences and outcomes (Holt et al., 2023). Addressing these disparities in health care requires policy, research, and practice changes, focusing on tailored health services that meet the unique needs of LGBTI individuals (Zeeman et al., 2019).

## Conclusion

From the unique biological and hormonal factors influencing sleep patterns to the multifaceted interplay between mental health, perceived gender discrimination (particularly in women of color) and other psychosocial factors with sleep quality, a holistic understanding of these complexities is crucial for sleep health providers. The advances in sleep disorder therapeutics over the past decade hold promise for improving the quality of life for women affected by these conditions. However, there is a prescient and ongoing need for further research, particularly in the field of sleep health disparities. Incorporating sex and gender differences in sleep research to accommodate the distinct biology of women is of the utmost importance (Mallampalli and Carter, 2014). The existing research gap regarding sleep disparities among diverse populations underscores the need for comprehensive studies utilizing consistent and robust measurement methods to investigate sleep characteristics and how they differ between diverse groups of people (Ahn et al., 2021). As the medical community continues to recognize the significance of sleep as a vital pillar of overall wellbeing, the integration of sex-specific considerations in research, diagnosis, and treatment strategies is essential to optimizing sleep health for women.

## Author contributions

EB: Writing – original draft. MP: Writing – review & editing. SJ: Writing – original draft, Writing – review & editing.
